# A recombinant conjugated pneumococcal vaccine that protects against murine infections with a similar efficacy to Prevnar-13

**DOI:** 10.1038/s41541-018-0090-4

**Published:** 2018-10-31

**Authors:** Mark Reglinski, Giuseppe Ercoli, Charlie Plumptre, Emily Kay, Fernanda C. Petersen, James C. Paton, Brendan W. Wren, Jeremy S. Brown

**Affiliations:** 1 Department of Respiratory Medicine, Centre for Inflammation and Tissue Repair, University College London, London, UK; 2 Department of Pathogen Molecular Biology, London School of Hygiene and Tropical Medicine, London, UK; 3 Department of Oral Biology, Faculty of Dentistry, University of Oslo, Oslo, Norway; 4 Department of Molecular and Biomedical Science, Research Centre for Infectious Diseases, The University of Adelaide, Adelaide, SA Australia

## Abstract

The pneumococcal conjugate vaccine (PCV) strongly protects against vaccine serotypes, but the rapid expansion of non-vaccine serotype disease and the vaccine’s high expense has reduced its overall impact. We have developed Protein Glycan Coupling Technology (PGCT) as a flexible methodology for making low-cost polysaccharide/protein glycoconjugates recombinantly in *Escherichia coli*. We have used PGCT to make a recombinant PCV containing serotype 4 capsular polysaccharide linked to the *Streptococcus pneumoniae* proteins NanA, PiuA, and Sp0148. The introduction of the *Campylobacter jejuni* UDP-glucose 4-epimerase gene GalE (*gne*) into *E. coli* improved the yield of the resulting glycoprotein. PGCT glycoconjugate vaccination generated strong antibody responses in mice to both the capsule and the carrier protein antigens, with the PiuA/capsule glycoconjugate inducing similar anti-capsular antibody responses as the commercial PCV Prevnar-13. Antibody responses to PGCT glycoconjugates opsonised *S. pneumoniae* and *Streptococcus mitis* expressing the serotype 4 capsule and promoted neutrophil phagocytosis of *S. pneumoniae* to a similar level as antisera generated by vaccination with Prevnar-13. Vaccination with the PGCT glycoconjugates protected mice against meningitis and septicaemia with the same efficacy as vaccination with Prevnar-13. In addition, vaccination with the protein antigen components from PGCT glycoconjugates alone provided partial protection against septicaemia and colonisation. These data demonstrate that a vaccine made by PGCT is as effective as Prevnar-13, identifies PiuA as a carrier protein for glycoconjugate vaccines, and demonstrates that linking capsular antigen to *S. pneumoniae* protein antigens has additional protective benefits that could provide a degree of serotype-independent immunity.

## Pneumococcal vaccines: evaluation of a multivalent pneumococcal glycoconjugate vaccine

The current pneumococcal conjugate vaccine induces protective immunity to infection, but this protection is limited to the dominant disease-causing strains included in the preparation. Emerging strains not included in the vaccine are not covered, which limits the vaccine’s protective effect in areas associated with alternative pneumococcal strains. Here Jeremy Brown, Brendan Wren and colleagues have optimised and applied Protein Glycan Coupling Technology to produce recombinant pneumococcal polysaccharide/protein glycoconjugates and evaluated these in a murine model of infection. Glycoconjugate vaccination resulted in antibody production specific to both capsule and the carrier proteins, which resulted in protection in murine models of meningitis and septicaemia equivalent to that of currently used vaccination strategies. This animal study highlights the potential of glycoconjugate vaccine approaches in the context of pneumococcal mediated disease.

## Introduction

*Streptococcus pneumoniae* (the pneumococcus) is a common cause of pneumonia, septicaemia, and meningitis, and consequently is responsible for a considerable burden of morbidity and mortality worldwide.^1^ *S. pneumoniae* meningitis is of particular concern owing to its high case fatality rate and the frequency of chronic neurological sequelae.^2^ The pneumococcal conjugate vaccine (PCV) is highly effective at preventing *S. pneumoniae* infections, including meningitis, caused by vaccine serotypes,^3–9^ but has important drawbacks. First, the dominant disease-causing serotypes (STs) vary geographically and with age group, yet the existing PCV formulation is fixed and not readily altered, and hence has a variable impact among different populations.^10^ Furthermore, PCV targets only 13 of the 90+ *S. pneumoniae* capsular STs, and PCV efficacy has been impaired by the major expansion of non-vaccine STs.^7,9,11–14^ Finally, PCV vaccines are produced by a multi-step chemical conjugation approach that involves hundreds of quality assurance steps that are expensive, restricting PCV use in low- and middle-income countries where the burden of disease is heaviest, and preventing the vaccine from being cost effective in adults.^15,16^ Overall, a low-cost *S. pneumoniae* PCV, which is flexible in antigen content to adjust for changes on *S. pneumoniae* ecology and provides a degree of ST-independent protection remains a global imperative.

We have pioneered a low-cost recombinant approach to making glycoconjugate vaccines termed Protein Glycan Coupling Technology (PGCT). PGCT uses a *C. jejuni* oligosaccharyltransferase, CjPglB, to link protein containing “glycotag” sequences to glycan structures that are co-expressed in *Escherichia coli.*^17–21^ Vaccine products are purified by a single step Ni^2+^ affinity chromatography procedure from *E. coli* cells grown in batch culture that can readily be scaled up for manufacture. Using PGCT to make PCV would be considerably simpler and have fewer quality control issues than existing chemical methodologies, resulting in cheaper vaccine with greater flexibility to alter ST content in response to the needs of different target populations or geographical locations, and facilitating rapid reformulation in response to changes in *S. pneumoniae* ecology. Another advantage of PGCT is that different protein antigens can be readily combined with capsular antigen. To date, only four major carrier proteins have been licensed for glycoconjugate vaccine formulations; deactivated toxins from *Clostridium tetanus* and *Corynebacterium diphtheria* (CRM_197_), and two surface expressed proteins from *Haemophilus influenzae* (Protein D) and *Neisseria meningitidis.*^22,23^ The efficiency of the antibody response to the glycan component of a glycoconjugate varies between peptides, yet the efficacy of multiple carrier proteins has not been tested limiting the development of glycoconjugate vaccines.^24,25^ Furthermore, using protective *S. pneumoniae* protein antigens as carrier proteins could provide ST-independent protection via antibody-mediated opsonophagocytosis,^26^ inhibition of bacterial protein function,^27,28^ and Th17 cellular immunity.^29–31^ Such a vaccine may also have theoretical advantages in preventing meningitis as antibodies to selected surface protein antigens could prevent penetration of the blood–brain barrier.^32–34^

PCGT has been used to make an effective prototype vaccine against *Francisella tularensis*^20^ and a *Shigella flexneri* PGCT vaccine that has completed phase one trials.^21^ We have shown PGCT can make recombinant *S. pneumoniae* capsular polysaccharides from four STs,^35^ but whether these capsular products can induce a similar level of protection as PCV has not been explored. These data are essential as proof of principle that the PGCT approach is a viable alternative to conventional manufacture of PCVs. Furthermore, whether *S. pneumoniae* protein antigens are effective carrier proteins for capsular antigens while simultaneously stimulating protective anti-protein immunity has not been investigated. To assess these gaps, we have tested in murine models the efficacy of a trivalent PCV made using PGCT to conjugate ST4 capsule to three *S. pneumoniae* protein antigens, an N-terminal fragment of NanA, a multifaceted virulence factor that promoted growth and survival in the nasopharyngeal tract, brain endothelial cell invasion, and synergistic infection with Influenza A,^32,36,37^ the Th17-stimulating antigen Sp0148^27^ and the ABC transporter lipoprotein PiuA.^38,39^ These antigens have previously been shown to be effective vaccine antigens in mouse models, and were chosen to specifically target prevention of meningitis or nasopharyngeal colonisation.

## Results

### The UDP-glucose 4-epimerase GalE improves glycoprotein production

**Fig. 1:**
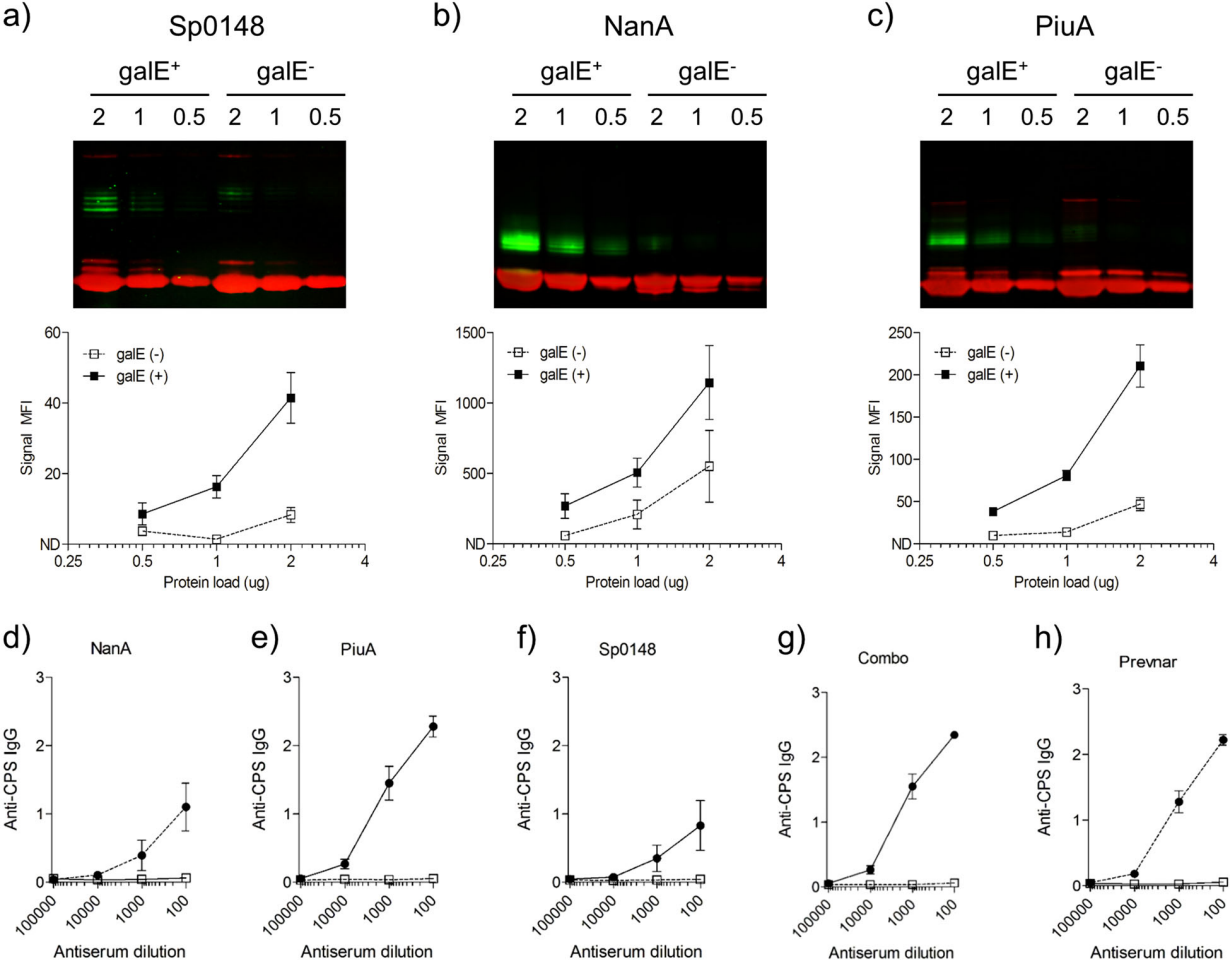
Analysis of recombinant proteins from glycoprotein expression strains. **a**-**c** Immunoblot analysis of decreasing concentrations of recombinant glycoproteins from *galE*^+^ and g*alE*^-^ *E. coli* strains. Fluorescent intensity of the signal from the glycosylated protein bands (green channel) was measured by densitometry using the LI-COR odyssey fluorescent imaging system and are displayed as mean ± SEM from three replicate experiments. Red channel: mouse anti-His IgG; Green channel: rabbit anti-Type 4 antiserum. **d**-**h** Anti-capsular polysaccharide antibody levels in antiserum measured from mice (*n* = 8) vaccinated with recombinant glycoproteins (closed circles) or cognate unglycosylated antigens (open squares). Antiserum from Prevnar-13 (closed circles) and PBS vaccinated (open squares) animals were included as controls. Results displayed as mean ± SEM from technical replicates

Using PGCT to produce recombinant glycoconjugates of *S. pneumoniae* ST4 capsule material linked to the *S. pneumoniae* protein antigens PiuA, Sp0148 and NanA initially resulted in relatively poor glycoconjugate yields (Fig. 1). We hypothesised that the intracellular availability of undecaprenyl phosphate carrier *N*-acetyl galactosamine (UDP-GalNac), the reducing end sugar required for ST4 capsule production, may be a limiting factor for ST4 capsule production by *E. coli.*^35^ Hence, a plasmid containing the gene encoding the *C. jejuni* UDP-glucose 4-epimerase GalE (*gne*), which reversibly converts UDP-GlcNac to UDP-GalNac^40^ was transformed into the PGCT *E. coli* strain. Densitometry of immunoblots demonstrated a marked increase in glycoconjugate yields from GalE^+^ *E. coli*, confirming that an increase in intracellular UDP-GalNac availability improved glycoprotein production (Figs. 1a-c). The intensity of glycoconjugate bands varied between the carrier proteins, with a more intense band seen for the NanA glycoconjugate, suggesting that glycosylation efficiency varies with the protein antigen used in the PGCT process.

### Recombinant PGCT glycoconjugates induces strong antibody responses against both the capsule and carrier protein antigens

**Fig. 2:**
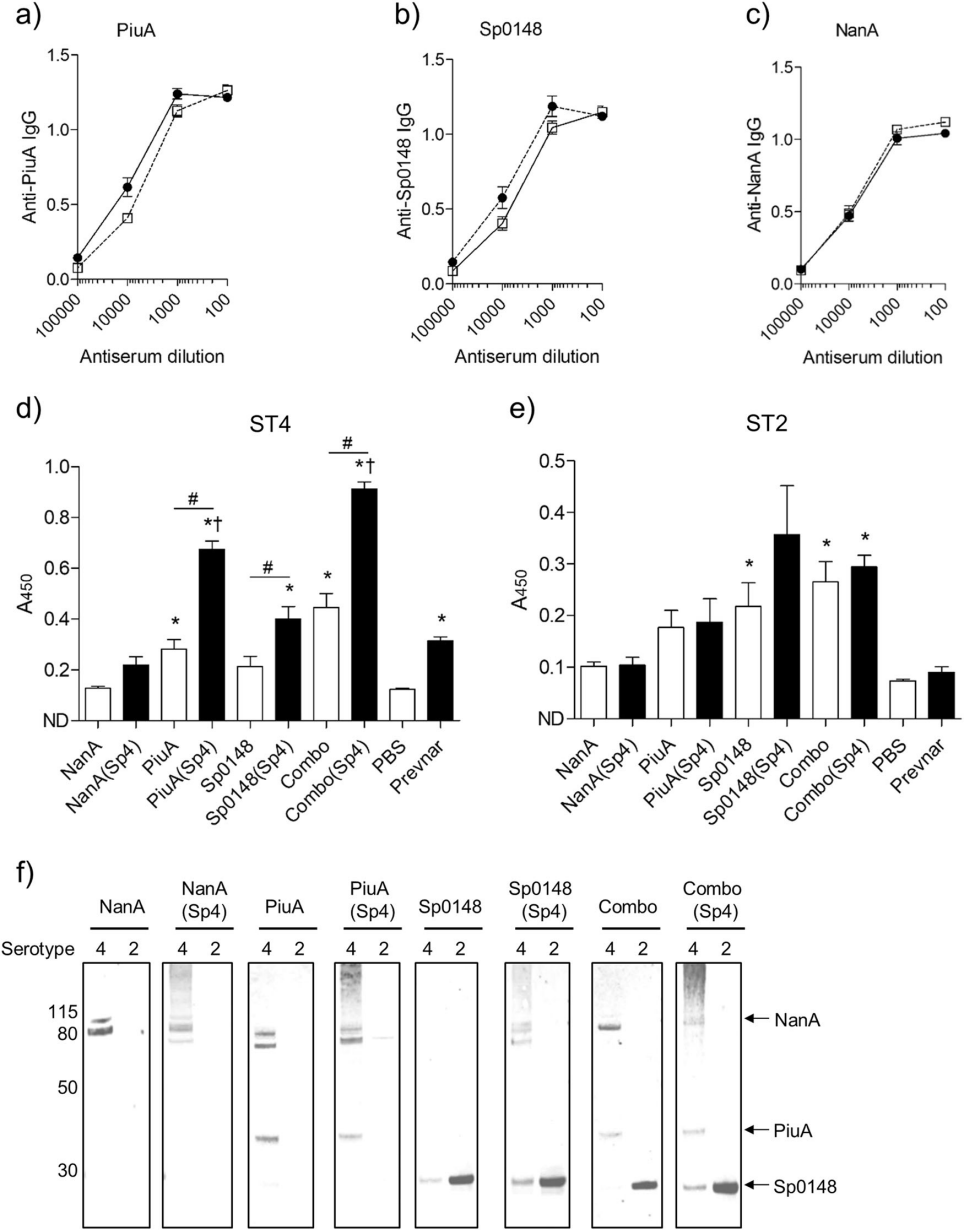
Vaccination with recombinant glycoproteins generates antibodies that recognise homologous and heterologous pneumococcal isolates. **a**-**c** Anti-carrier protein antibodies were measured from mice vaccinated with Combo(Sp4) (closed circles) or Combo (open squares) by sandwich ELISA using a monoclonal anti-His capture antibody and recombinant, unglycosylated carrier proteins. Data are displayed as mean ± SEM from technical replicates. **d**, **e** Anti-ST4 **d** and anti-ST2 **e** antibodies were measured by whole-cell ELISA using pooled antiserum from the glycosylated (black bars) and unglycosylated (white bars) vaccine groups. Data are displayed as mean ± SEM from three separate cultures. *p < 0.05 vs PBS, ^#^*p* < 0.05 protein vs glycoprotein, ^†^*p* < 0.05 vs Prevnar-13 one-way ANOVA with Bonferroni’s post-test. **f** Immunoblot analysis of concentrated lysate from ST4 and ST2 overnight cultures using pooled antiserum from the glycosylated and unglycosylated vaccine groups. Molecular mass markers are given in kilodaltons

Mice were vaccinated with PGCT glycoconjugates NanA(Sp4), PiuA(Sp4), and Sp0148(Sp4) prepared from GalE^+^ isolates using a three-dose schedule, with cognate unglycosylated antigens (both singly and in combination, labelled Combo), Prevnar-13, and phosphate buffered saline (PBS)/adjuvant vaccine groups included as controls. Serum anti-capsule antibodies were above the limit of detection in all recombinant PGCT glycoconjugate groups, with no reactivity detected in groups vaccinated with protein alone (Figs. 1d-h). Anti-capsule antibody levels varied between groups, but did not correlate with the relative levels of glycoprotein present. Although the highest level of glycosylation occurred with the NanA protein (Fig. 1a), PiuA glycoconjugates stimulated the most robust anti-capsular immune responses (Fig. 1e). Vaccination with Combo(Sp4), a combination of all three glycoconjugates, generated a strong anti-capsule response similar to the response to Prevnar-13. A sandwich enzyme-linked immunosorbent assay (ELISA) confirmed that all vaccine groups had good antibody responses to the carrier proteins, with no evidence that glycosylation impaired protein antigenicity (Figs. 2a-c).

### Antibody recognition of *S. pneumoniae* in sera from mice vaccinated with recombinant glycoconjugates

Antibody recognition of *S. pneumoniae* by sera from vaccinated mice was assessed by whole-cell ELISA using the homologous *S. pneumoniae* ST4 strain TIGR4 or the heterologous ST2 strain D39 (Figs. 2d, e). Sera from mice vaccinated with the PGCT glycoconjugate PiuA(Sp4) or all three PGCT glycoconjugate, Combo(Sp4), had significantly higher anti-TIGR4 titres than mice vaccinated with Prevnar-13 (Fig. 2d). Although the response in the Sp0148(Sp4) vaccine group was lower than that generated by Prevnar-13, the anti-TIGR4 titre in these animals was significantly higher than in the PBS group. Mice vaccinated with the NanA glycoconjugate generated a nonsignificant increase in anti-TIGR4 titres compared with PBS vaccinated animals. Significant anti-TIGR4 titres were also recorded following vaccination with unglycosylated PiuA and the combination of all three unglycosylated proteins, indicating that the anti-protein antibody response recognised *S. pneumoniae*. As expected, anti-D39 responses did not differ between glycoconjugate and protein only vaccine groups with a significant increase in antibody titre to D39 in mice vaccinated with Sp0148, Sp0148(Sp4), or the combination of all three proteins alone, and nonsignificant increases for the remaining PGCT vaccine groups (Fig. 2e).

Recognition of natural pneumococcal antigens was confirmed by immunoblotting *S. pneumoniae* lysates (Fig. 2f). Probing the TIGR4 lysates with sera from mice vaccinated with PGCT glycoconjugates resulted in a diffuse high molecular weight signal that represented capsule material. The immunoblot signal for capsular material was weaker for TIGR4 lysates probed with sera from Sp0148(Sp4) and NanA(Sp4) vaccinated mice, compatible with the results of the capsule ELISAs (Figs. 1d-h), and as expected was absent for the D39 lysates. Bands of the expected molecular weight for NanA, Sp0148, and PiuA were present when TIGR4 lysates were probed with the corresponding antisera (Fig. 2f). Consistent with the results of the whole-cell ELISAs, probing D39 lysates resulted in a good signal representing Sp0148 (stronger than for the TIGR4 lysates), a weak signal for PiuA, and no detectable signal representing NanA. Together these data indicate that vaccination with the PGCT glycoconjugates generated antibodies to both the ST4 capsule and protein antigens, but recognition of the latter varied between *S. pneumoniae* strains.

### Antibodies induced by recombinant glycoconjugates opsonised live *S. pneumoniae*

**Fig. 3:**
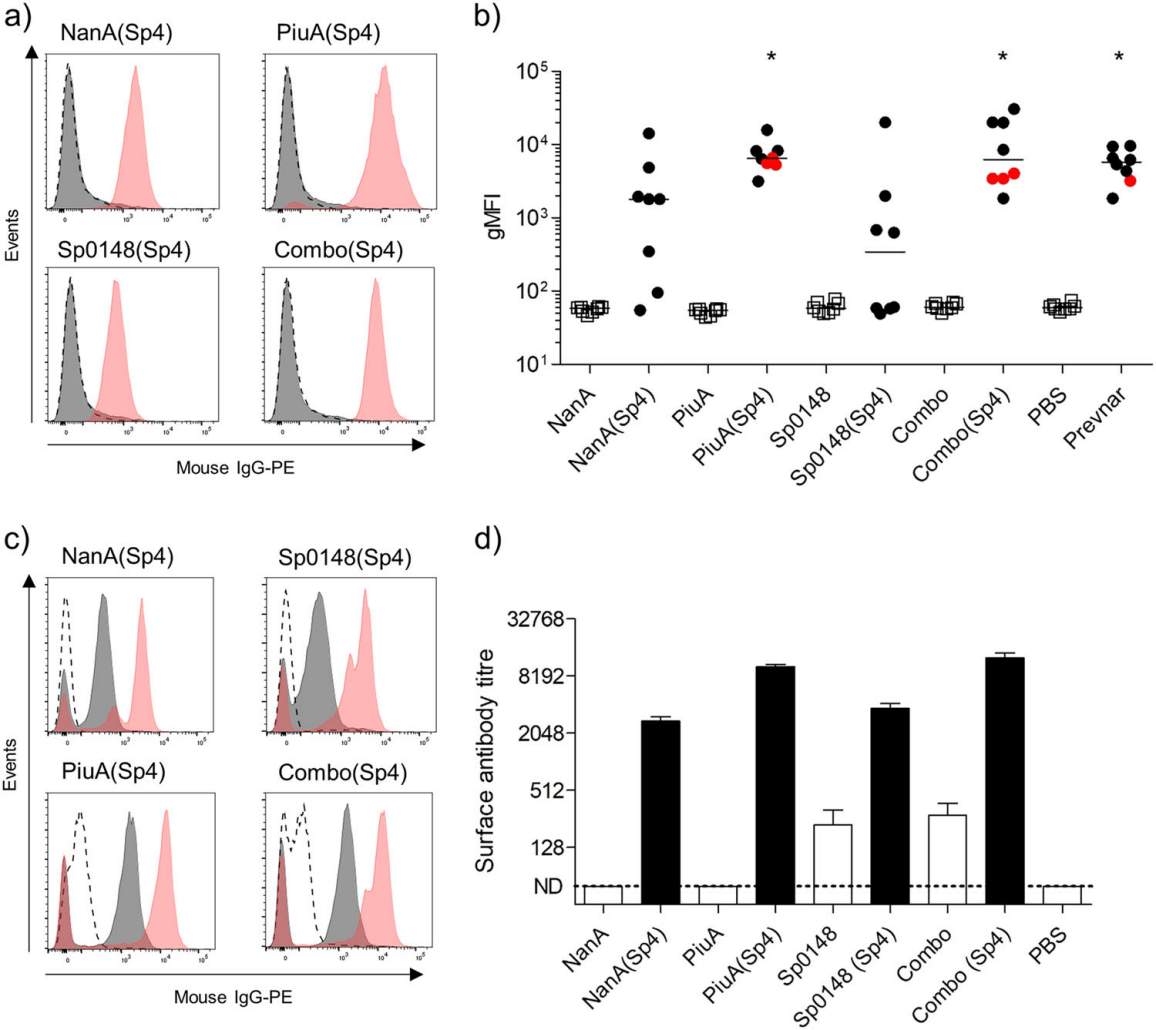
Flow cytometry analysis of antibody deposition on streptococcal species. **a** Representative histograms for antibody deposition on *S. mitis*(SpT4) in 10% antiserum from glycosylated (red shading) and unglycosylated (grey shading) vaccine groups. PBS vaccinated serum (dashed line) was included as a control. **b** Antibody deposition measured using a flow cytometry assay on *S. mitis*(SpT4) in 10% murine antiserum (*n* = 8) from glycosylated (closed circles) and unglycosylated (open squares) vaccine groups. Red dots indicate reactions containing reduced antiserum concentrations (5 vs 10%) in high titre samples. *p < 0.05 Kruskal–Wallis with Dunn’s post-test (vs PBS). **c** Examples of flow cytometry histograms for antibody deposition on the TIGR4 *S. pneumoniae* strain in 2% (red shading), 0.2% (grey shading), and 0.02% (dashed line) antiserum from glycosylated vaccine groups. **d** Antibody deposition on TIGR4 in pooled antiserum from mice vaccinated with glycosylated or unglycosylated vaccines. Deposition titres were determined using bacteria incubated with decreasing concentrations of Prevnar-13 antiserum to generate a standard curve. Results displayed as mean ± SEM from technical replicates

Flow cytometry assays were used to assess whether antibody induced by vaccination with PGCT glycoconjugates can opsonise live *S. pneumoniae*. To specifically investigate anti-capsular recognition, we assessed IgG binding to a *S. mitis* mutant expressing the *S. pneumoniae* ST4 capsule (*S. mitis*(SpT4)).^41^ No recognition of wild-type *S. mitis* was detected (Figure S2), but IgG in sera from mice vaccinated with each of the PGCT glycoconjugates or Prevnar-13 bound to *S. mitis*(SpT4) (Figs. 3a, b). The degree of IgG binding varied between vaccine groups, with *S. mitis*(SpT4) recognition in sera from only four and six mice vaccinated with Sp0148(Sp4) or NanA(Sp4), respectively, whereas sera from all eight mice vaccinated with PiuA(Sp4) or Combo(Sp4) caused significant IgG binding to *S. mitis*(SpT4) at a comparable level to that seen for the Prevnar-13 vaccinated group (Fig. 3b). No antibody deposition on the *S. mitis*(SpT4) strain was seen in sera from mice vaccinated with unglycosylated protein antigens, confirming a lack of cross reactivity between the pneumococcal carrier proteins and the *S. mitis* cell surface. These data combined with the data from Figs. 1 and 2 confirm that the PiuA(Sp4) glycoconjugate induced an anti-capsular antibody response that was similar in strength to Prevnar-13 and was significantly stronger than the response to the NanA(Sp4) and Sp0148(Sp4) glycoconjugates.

**Fig. 4:**
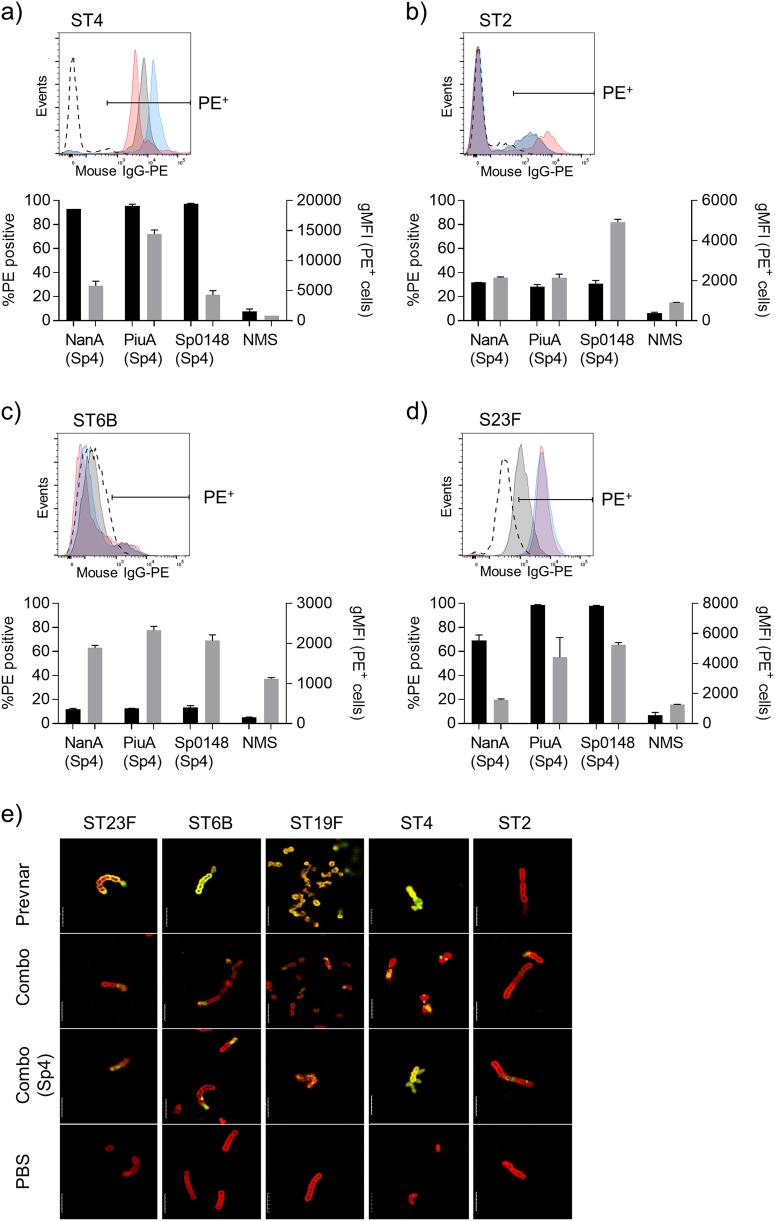
Antibody deposition on non-serotype 4 pneumococci. **a**-**d** Representative histograms and antibody deposition on homologous and heterologous pneumococcal isolates in 1% pooled antiserum from mice vaccinated with glycosylated NanA (grey shading), Sp0148 (red shading), or PiuA (blue shading) or normal mouse serum (dashed line). Black bars represent the percentage of PE^+^ bacteria and grey bars represent the gMFI of the positive population. Gates were set such that 5–10% of events were PE^+^ in the normal mouse serum (NMS) reactions to account for strain specific differences in auto fluorescence. Data are displayed as mean ± SEM from technical replicates. **e** Immunofluorescent staining of homologous and heterologous pneumococcal isolates using antiserum from mice vaccinated with the combination vaccine (green channel) and pneumococcal Omni serum (red channel). Length of scale bar is equal to 5 µm

To assess opsonisation of *S. pneumoniae*, the flow cytometry IgG-binding assays were repeated using different *S. pneumoniae* strains. Incubation of TIGR4 with descending dilutions of pooled sera from mice vaccinated with PGCT glycoconjugates demonstrated dose-dependent IgG binding (Fig. 3c). Comparison of the geometric mean fluorescence intensity (gMFI) readings with a standard curve generated using antiserum from Prevnar-13 vaccinated mice revealed high levels of IgG binding to *S. pneumoniae* in serum from mice vaccinated with the individual glycoconjugates and Combo(Sp4) (Fig. 3d). In addition, there were detectable levels of surface IgG binding in sera from mice vaccinated with unglycosylated Sp0148 or all three unglycosylated proteins combined (Fig. 3d), but not for sera obtained from unglycosylated NanA or PiuA. To assess recognition of the individual protein antigens, IgG binding to other capsular STs of *S. pneumoniae* was assessed and presented as the proportion of bacteria positive for surface IgG and the intensity of IgG binding on the positive bacteria (Figs. 4a-d). IgG recognition was generally weaker than for the TIGR4 strain and varied between strains, with good levels of IgG binding to the ST23F strain, some binding to the ST2 strain, but little binding to the ST6B strain. The ability of each protein antigen to promote IgG binding also varied between STs; for example, NanA induced the weakest responses against the ST23F strain, and Sp0148 for the TIGR4 strain. This perhaps reflects differences between strains in the expression level and surface accessibility of individual protein antigens.

### Immunofluorescence of IgG binding to *S. pneumoniae* in sera from PGCT glycoconjugate vaccinated mice

Recognition of homologous and heterologous pneumococcal isolates was further investigated by fluorescence microscopy (Fig. 4e). Incubation in sera from Prevnar-13 vaccinated mice caused bright, uniform surface staining for all the vaccine STs tested (ST4, ST6B, ST19F, and ST23F) but not the non-vaccine ST2 strain D39. When incubated in sera from mice vaccinated with Combo(Sp4), only the TIGR4 strain had a similar level of bright, uniform staining across the bacterial surface. However, a weaker and patchy pattern of staining was seen for all STs incubated in sera from mice vaccinated with either the glycosylated Combo(Sp4) or unglycosylated Combo of all three protein antigens (Fig. 4e). Together the ELISA, flow cytometry, and immunofluorescence data demonstrate that PGCT glycoconjugates induced anti-capsular IgG responses that varied in strength between carrier proteins, but were similar in strength for PiuA glycoconjugates to those induced by Prevnar-13. In addition, the PGCT glycoconjugates stimulated antibody responses to protein antigens that partially opsonised heterologous *S. pneumoniae* STs (Fig. 4) and therefore potentially provide some degree of ST-independent protection.

### Ability of sera from mice vaccinated with recombinant PGCT glycoconjugates to support neutrophil phagocytosis

**Fig. 5:**
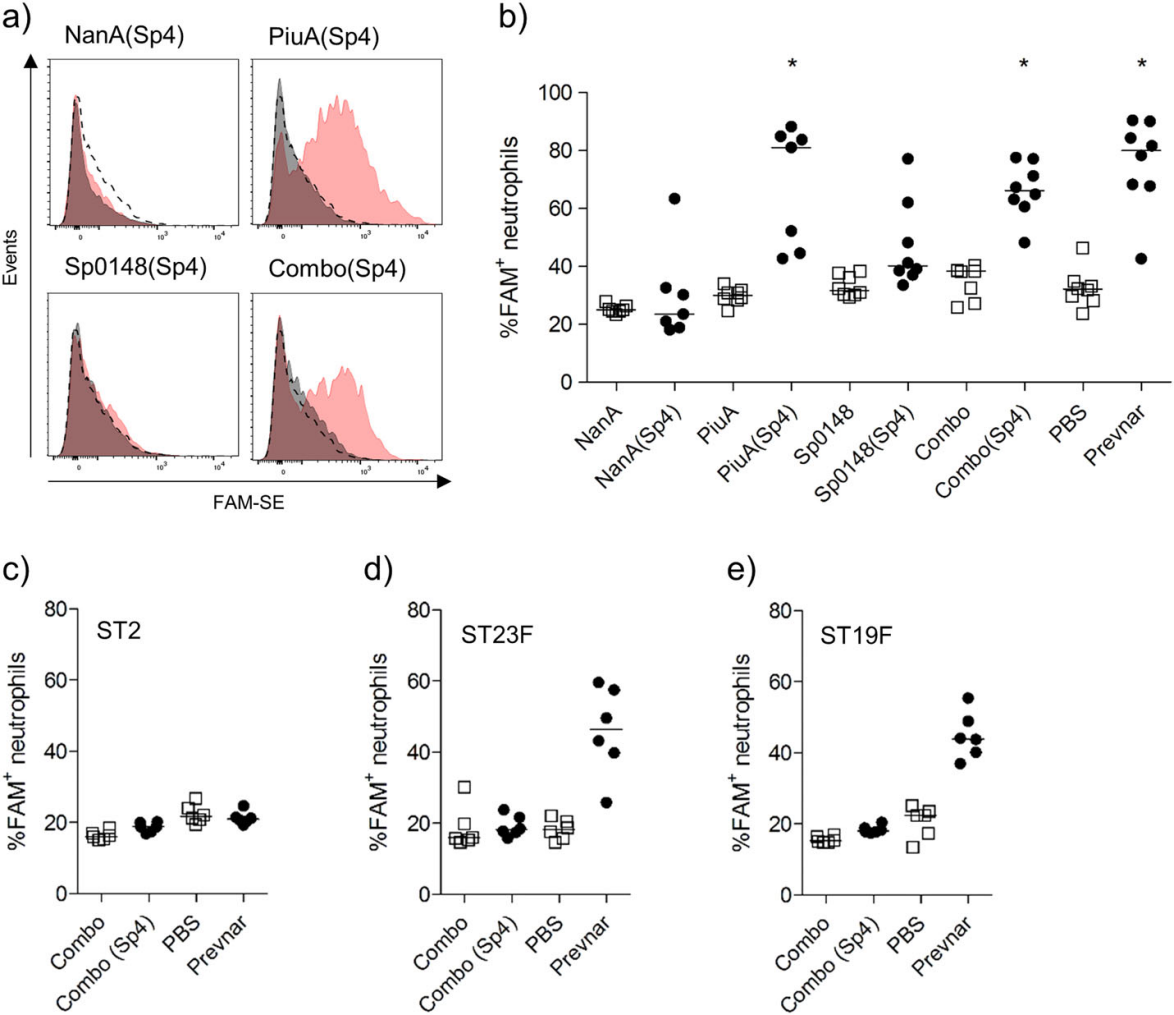
Effect of antiserum samples on interaction of *S. pneumoniae* with human neutrophils. **a** Examples of flow cytometry histograms for fresh human neutrophils incubated with FAM-SE labelled TIGR4 when opsonised in 20% antiserum from glycosylated (red shading) or unglycosylated (grey shading) vaccine groups and 5% baby rabbit complement. PBS vaccinated serum (dashed line) was included as a control. **b** Percent association of fresh human neutrophils with TIGR4 when opsonised in 20% antiserum (*n* = 8) from glycosylated (closed circles) or unglycosylated (open squares) vaccine groups and 5% baby rabbit complement. Antiserum from Prevnar-13 (closed circles) and PBS vaccinated (open squares) animals were included as controls. *p < 0.05 Kruskal–Wallis with Dunn’s post-test (vs PBS). **c**-**e** Percent association of fresh human neutrophils with non-type 4 pneumococci when opsonised in 20% antiserum (*n* = 6) from glycosylated (closed circles) or unglycosylated (open squares) vaccine groups and 5% baby rabbit complement. Antiserum from Prevnar-13 (closed circles) and PBS vaccinated (open squares) animals were included as controls

To determine if antibody responses to PGCT glycoconjugates can promote opsonophagocytosis, neutrophil uptake assays were performed.^42^ Incubation in sera from mice vaccinated with the PiuA(Sp4) glycoconjugate or Combo(Sp4) promoted neutrophil uptake of the TIGR4 *S. pneumoniae* strain (Fig. 5a). In contrast, sera from mice vaccinated with the Sp0148(Sp4), NanA(Sp4), or the unglycosylated antigens (individually and in combination) failed to promote neutrophil phagocytosis in this assay (Fig. 5b), and none of the sera from any of the PGCT glycoconjugate vaccine groups promoted neutrophil uptake of three heterologous STs (ST2, ST23F, and ST19F) (Figs. 5b-e). These data confirm that recombinant PGCT type 4 capsular antigen can induce functionally important IgG, but the relatively patchy and low level of IgG binding to the protein antigens alone was not sufficient to promote neutrophil phagocytosis in the conditions used for this assay.

### Protective efficacy of vaccination of mice with recombinant glycoconjugates produced by PGCT

**Fig. 6:**
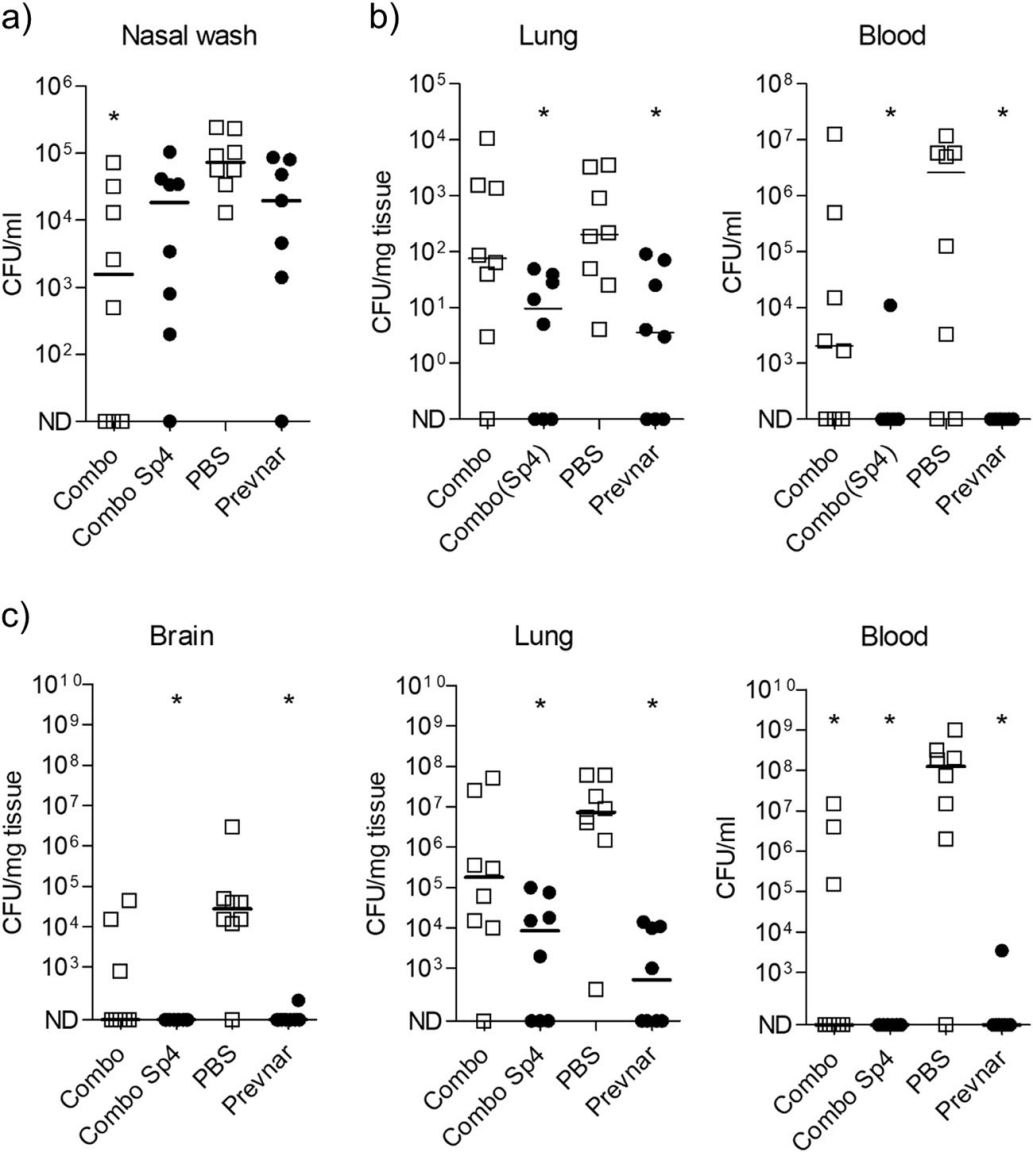
Vaccination with recombinant glycoconjugates provides protection against several pneumococcal disease aetiologies. Mice were vaccinated with Combo(Sp4), Combo, Prevnar-13, or PBS+ adjuvant as outlined in the methods. **a** Mice challenged intranasally with 1 × 10^7^ CFU of TIGR4 were culled seven days post-infection and bacterial loads were assessed by nasal washing. **b**, **c** Mice were infected intranasally with 1 × 10^7^ CFU of TIGR4 **b** or 2 × 10^6^ CFU of WCH43 **c** and culled after 24 or 48 h respectively. Bacterial burdens in the blood and organ homogenates were determined by serial dilution and plating. *p < 0.05 Kruskal–Wallis with Dunn’s post-test (vs PBS)

Mouse models of colonisation, pneumonia with sepsis, and meningitis were used to assess the protective efficacy of vaccination with Combo(Sp4) or Combo, using Prevnar-13 as a positive control. In the model of TIGR4 colonisation, mice vaccinated with the Combo had reduced TIGR4 colony forming units (CFU) in nasal washes recovered seven days after initial colonisation (Fig. 6a). In the Prevnar-13 and Combo(Sp4) vaccine groups, there was an approximately 1-log_10_ reduction in nasal wash CFU compared with the control group, but these differences were not statistically significant. In the TIGR4 pneumonia with septicaemia model, vaccination with the Combo(Sp4) almost completely prevented septicaemia and resulted in an approximately 1-log_10_ reduction in lung CFU, a similar level of protection to that provided by Prevnar-13 (Fig. 6b). Although not statistically significant, there was also a reduction in median CFU/ml recovered from the blood in mice vaccinated with the combination of the unglycosylated proteins. In the meningitis model, mice were given an intranasal inoculation of WCH43, a ST4 isolate known to cause meningitis in a murine model.^34^ In this model, vaccination with Combo(Sp4) again resulted in a similar level of protection to that provided by vaccination with Prevnar-13, with complete prevention of septicaemia and meningitis and an approximately 3-log_10_ reduction in lung CFU (Fig. 6c). For mice vaccinated with Combo (proteins alone), median blood, lung, and brain CFU were all lower than results for the negative control although only the blood data were statistically significant (Fig. 6c). Overall, these data demonstrate that the PGCT glycoconjugate vaccine was protective against infection with the homologous *S. pneumoniae* ST, providing a level of protection as good as vaccination with Prevnar-13. Furthermore, the *S. pneumoniae* protein components generated some protective responses independent of capsular antigen, suggesting that they may provide a degree of ST-independent protection.

## Discussion

Despite the efficacy of the PCV, *S. pneumoniae* remains a major pathogen driving worldwide infectious mortality and morbidity. Extension of routine vaccination with PCV to all countries will be necessary to reduce this substantial disease burden, but this is prevented by the current high cost of chemically coupled preparation of PCVs. Furthermore, routine use of PCV drives ST replacement leading to a probable reduction in vaccine efficacy over time. Thus, there is an urgent need to reduce PCV production costs, increase the flexibility of PCV manufacture to allow additional STs to be introduced in response to changes in *S. pneumoniae* epidemiology, and preferably improve the vaccine to include additional non-ST-dependent immunity. In this report, we demonstrate recombinant *S. pneumoniae* glycoconjugates expressed in *E. coli* cells using PGCT can fulfil these requirements, making PGCT a viable alternative methodology for the manufacture of a new generation of PCVs. Our major findings are: (a) *S. pneumoniae* capsular material made in *E. coli* and linked to a carrier protein using PGCT stimulates anti-capsular antibodies; (b) anti-capsular immunogenicity of PGCT glycoconjugates varies between carrier proteins, with at least one *S. pneumoniae* protein antigen (PiuA) stimulating anti-capsular antibody levels similar to those induced by a commercial PCV; (c) the carrier proteins are also immunogenic, stimulating antibody responses able to offer some degree of protection against colonisation, meningitis, and sepsis independent of capsular antibody; and (d) that in mouse models of colonisation, pneumonia, and meningitis vaccination with our PGCT glycoconjugate provides a similar level of protection as Prevnar-13. An additional finding is that glycoconjugate yield and PGCT efficiency can be improved by expressing a UDP-glucose 4-epimerase, GalE, in the *E. coli* host strain.

Our data have been obtained using a single capsular ST, but to be a viable methodology for manufacturing PCV the number of STs will have to be expanded to match the up to 13 STs included in existing PCVs. Several other *S. pneumoniae* capsule STs have been expressed in *E. coli* and coupled to a variety of carrier proteins using the *C. jejuni* (Wren, unpublished data). However, the *C. jejuni* PglB can only transfer polysaccharides ending in an acetylated sugar,^43^ and thus is suitable for only 12 STs (1, 4, 5, 12F, 12A, 12B, 25F, 25A, 38, 44, 45, and 46). To overcome this problem, additional bacterial oligosaccharyltransferases with different sugar specificities,^44,45^ or with more promiscuous recognition of glycans^46^ are being actively developed for PGCT to allow production of glycoconjugates that include the majority of vaccine STs, as well as vaccine-replacement STs. Creating a range of *E. coli* cells expressing different *S. pneumoniae* STs will allow the ST content of a PGCT PCV to be altered rapidly in response to changes in ST prevalence or tailored to specific target populations, a major additional advantage over PCVs made using conventional chemical conjugation methodologies.

The presented data demonstrate that glycoconjugates produced by PGCT are capable of inducing an equivalent anti-capsular antibody response to Prevnar-13 that is highly protective in the pneumonia model, causing almost complete prevention of septicaemia. As a consequence, any additional benefit against ST4 *S. pneumoniae* strains of the anti-protein response to vaccination with PGCT PCV would be hard to detect, and require mouse group sizes that are ethically unjustified. Instead, the main clinical benefit of anti-protein responses will be in extending protection to heterologous STs. In addition, making PCVs using PGCT rather than by chemical reaction still has considerable practical advantages in reducing cost and complexity of the manufacturing process. Although our results for protection against colonisation showed the PGCT PCV reduced nasopharyngeal CFU at 7 days these data did not reach statistical significance. The results for the PGCT PCV were similar to those for Prevnar-13, which despite the known effects of Prevnar-13 in preventing colonisation in humans also did not show statistical significant reductions in CFU compared with the controls. Additional experiments with an earlier or later timepoint or a larger *n* number are necessary to fully define the effects of the PGCT PCV on nasopharyngeal carriage.

A potential important advantage of using *S. pneumoniae* protein antigens as carrier proteins for a PCV made using PGCT is the induction of protective ST-independent anti-protein immunity that could include both humoral immunity and Th17 responses, which are thought to enhance mucosal immunity compared with antibody responses alone.^27,47,48^ We have investigated this using three conserved *S. pneumoniae* protein antigens known to be protective in vivo*;*^27,49^ NanA, chosen as it is required for *S. pneumoniae* to cross the blood–brain barrier and hence anti-NanA could specifically prevent meningitis;^32^ Sp0148, chosen as it is a recognised Th17 antigen that promotes mucosal immunity;^27^ and PiuA, chosen as it is highly expressed by *S. pneumoniae* colonising the nasopharynx so could improve prevention of colonisation.^39^ Our data confirmed that vaccination with PGCT glycoconjugates containing NanA, PiuA, and Sp0148 carrier proteins generated strong anti-protein antibody responses. However, as previously recognised with other protein antigens, these anti-protein antibody responses had weaker opsonising potential than anti-capsular antibody, and was unable to support in vitro neutrophil phagocytosis under the test conditions we used. This probably reflects the lower quantities of expression of surface proteins compared with capsular antigen and their protection from antibody recognition by the capsule.^50^ Furthermore, using flow cytometry and immunofluorescence we have shown that despite high levels of conservation (at least for Sp0148 and PiuA), the strength of antibody recognition of each *S. pneumoniae* protein antigen differs between *S. pneumoniae* strains. This probably reflects differences in levels of protein expression between strains, and also differences between capsular STs in allowing antibody access to subcapsular protein antigens.^51^ Repeat IgG opsonisation experiments with antisera to individual carrier proteins could help identify which protein dominates the response to different heterologous strains. Antiserum raised against Sp0148 was shown to cross react with TIGR4 and D39 by whole-cell ELISA, confirming that this antigen is expressed at high enough levels to promote recognition in some settings. The ability of Sp0148 to generate cross reactive antiserum is supported by data from a recent global study predicting that inclusion of the TIGR4 variant of Sp0148 in a multivalent protein vaccine would potentially provide 98% coverage against ST1 pneumococcus worldwide.^52^ Interestingly, anti-protein immunity alone did cause a statistically significant reduction in nasopharyngeal colonisation, which could suggest these antigens can induce a Th17 response similar to that described previously for Sp0148.^27^

However, protein antigen selection will need to take into account not only their degree of conservation between strains but also variation in expression levels and accessibility to antibody, factors that cannot easily be predicted using bioinformatics. Indeed, recognition of the selected proteinaceous virulence factors may vary drastically in vivo where capsule and virulence protein expression may be modulated in response to a variety of external stimuli. Although it is likely that increased capsule expression will make recognition of subcapsular antigens in the blood more difficult, the effects in the nasopharynx and blood–brain interface are harder to predict.

Despite the limited effects of antibody targeting NanA, Sp0148, and PiuA in supporting neutrophil phagocytosis, vaccination with these proteins alone reduced bacterial CFU present in blood and meninges after challenge with *S. pneumoniae*. These data demonstrate the protective potential of including *S. pneumoniae* carrier proteins in PGCT glycoconjugates, although the weaker protective effects of anti-protein compared with anti-capsular antibody demonstrate a need to identify additional *S. pneumoniae* proteins able to induce stronger cross-protective immunity against multiple *S. pneumoniae* strains.

The ELISA, flow cytometry, and fluorescence microscopy data all demonstrate that PiuA is a highly effective carrier protein option for *S. pneumoniae* glycoconjugate vaccines, and therefore possibly for other non-pneumococcal glycoconjugates. This is a major finding that has wider implications for vaccine development as these results suggest PiuA could be an additional carrier protein for glycoconjugate vaccines to add to the four existing proteins. Sp0148 and NanA were considerably less effective at generating anti-capsular antibody responses than PiuA, and it probably most of the anti-capsule to the Combo(Sp4) vaccine resulted from the glycosylated PiuA component. The reasons why there were such marked differences in the efficacy of the carrier protein in promoting anti-capsular antibody is not clear. Immunoblots suggested that this is not simply due to a greater quantity of PiuA glycoconjugate, as the NanA glycoconjugate seemed to be the most abundant (Fig. 1). Recent data suggest glycoconjugates generate an antibody response to glycan by presentation of a glycosylated protein epitope bound to MHCII directly to the T-cell receptor.^24,25^ As some protein epitopes will be presented more efficiently, this mechanism would be affected by the protein carrier and perhaps by the site of covalent linkage to capsular antigen and therefore explain our data. Screening multiple additional *S. pneumoniae* proteins should identify additional suitable carrier proteins for glycoconjugate vaccines.

In summary, the data presented here demonstrate that a recombinant *S. pneumoniae* protein/capsular antigen glycoconjugate made by PGCT has a similar efficacy to a commercial PCV in preventing ST4 infection, but can also induce a degree of anti-protein protective immunity. With further development to expand the STs compatible with PGCT and to identify the most effective cross-protective *S. pneumoniae* carrier protein antigens, PCVs made using PGCT could provide a low-cost flexible method of manufacture that will make PCVs affordable to low- and middle-income countries.

## Materials and methods

### Bacterial strains and growth conditions

The bacterial strains used in this study are listed in Table S1. *E. coli* isolates were cultured in modified super optimal broth (SOB)^35^ or agar at 28 °C, supplemented with 100 µg/ml ampicillin, 20 µg/ml tetracycline, and/or 80 µg/ml spectinomycin when appropriate. *S. pneumoniae* were cultured on Columbia horse blood agar plates (E&O laboratories) or in brain heart infusion broth at 37 °C + 5% CO_2_, supplemented with 5 µg/ml gentamycin or 75 µg/ml streptomycin when appropriate. *S. mitis* was cultured in Todd–Hewitt broth supplemented with 0.5% yeast extract at 37 °C + 5% CO_2._ *S. pneumoniae* and *S. mitis* were cultured to an OD_600_ of approximately 0.4–0.8 and stored in single use 1 ml aliquots at −80 °C in 20% glycerol.

### Synthesis and genetic modification of carrier protein genes

DNA sequences, codon optimised for expression in *E. coli*, encoding neuraminidase A (NanA, Spd1504) and the ABC transporter proteins PiuA (Sp1872) and Sp0148 were synthesised commercially in pUC57 and sub-cloned into pEXT21 as outlined in the supplementary methods (Table S2). Owing to the large size of full-length NanA, which reduced recombinant protein yields, the N-terminal lectin-like domain of the protein (essential for protein function^32^) was sub-cloned as outlined in the supplementary methods. Gene sequences for PiuA and Sp0148 were derived from the genome of the TIGR4 strain, whereas the gene sequence for NanA was derived from the genome of the D39 strain.

### Preparation of expression strains

*E. coli* W3110 and W311B pB4-4 cell cultures were inoculated 1:100 from overnights and grown to an OD_600_ of 0.3–0.6, chilled on ice, pelleted at 4000 × *g* for 10 min and washed sequentially with 0.5 volumes and then 0.25 volumes of ice cold 10% glycerol. Competent cells were resuspended in 1/250^th^ volume ice cold 10% glycerol and 50 µl aliquots were transformed with pEXT21(*nanA*), pEXT21(*piuA*), or pEXT21(*sp0148*) in a 0.2 cm gap cuvette at 2.5 kV, 200 Ω, and 25 µF. The *C. jejuni* UDP-glucose 4-epimerase GalE (*gne*) was amplified from pEXT21(*gne*) using the pEXT-F/pEXT-R primer pair and sub-cloned into pEXT20 using the restriction enzymes *Eco*RI and *Pst*I (New England Biolabs), before transformation into W311B pB4-4 pEXT21(*nanA/piuA/sp0148*) by electroporation as outlined above. Isolates were stored at −80 °C in 20% glycerol.

### Recombinant protein preparation

W3110 pEXT21(*nanA/piuA/sp0148*) and W311B pB4-4 pEXT20(*galE*) pEXT21(*nanA/piuA/sp0148*) isolates were cultured overnight at 28 °C and sub cultured 1:100 into SOB for 2–3 h prior to overnight induction with 1 mM isopropyl β-D-1-thiogalactopyranoside (IPTG) and 4 mM MnCl_2_ at 28 °C. The cells were pelleted at 14,000 × *g* and lysed using a pressure cell homogeniser (Stanstead). Lysates were treated with 25 U/ml Benzonase Nuclease (Sigma-Aldrich) for 20 min at RT and 0.2 µm filtered using Millex-GP Syringe Filters (Millipore). In initial studies, recombinant (glyco)proteins were purified using the Ni-NTA purification system (Thermo Fisher Scientific). For vaccination studies, recombinant (glyco)proteins were isolated using GE Healthcare His-trap FF columns and an AKTA purifier with a linear imidazole gradient of 25–250 mM. Sample purity was confirmed by sodium dodecyl sulfate polyacrylamide gel electrophoresis (SDS-PAGE) (Figure S1).

### SDS-PAGE and immunoblotting

SDS-PAGE and protein transfer was performed using NuPAGE Bis-Tris protein gels and the iBlot2 transfer system according to the manufacturer’s instructions (Thermo Fisher). Immunoblotting was performed under standard conditions using ST4 rabbit anti-capsule antibody (1:1000, Statens Serum Institut, Denmark), monoclonal mouse anti-His IgG (1:5000, Abcam), and detection with 1:10,000 dilutions of goat anti-rabbit IgG (IRDye800) and goat anti-mouse IgG (IRDye680) and the LI-COR odyssey fluorescent imaging system and densitometry (LI-COR Biosciences UK Ltd). TIGR4 and D39 lysates were prepared by pressure lysis of overnight cultures, 0.2 µm filtered and concentrated using Vivaspin 20 Centrifugal Concentrators (10,000 MWCO). In all, 10 µl aliquots of concentrated lysate were analysed by immunoblotting using a 1:1000 dilution of murine antiserum and a 1:10,000 dilution of goat anti-mouse IgG (IRDye800). All blots derived from the same experiment were processed in parallel.

### Vaccination studies

For antiserum generation and pneumonia/meningitis challenge studies, female 5- to 6-week CD1 mice (Charles River) were vaccinated on day 0 intraperitoneally with recombinant (glyco)proteins singly (10 µg/mouse/injection) or in combination (Combo/Combo(Sp4), 4 µg each protein/mouse/vaccination) emulsified 1:1 in Sigma adjuvant (Sigma-Aldrich), 20 µl of Prevnar-13 (Wyeth) diluted 1:5 PBS, or PBS and Sigma adjuvant alone. Booster immunisations were given subcutaneously on days 21 and 35. In pneumonia and meningitis infection experiments, mice were infected intranasally with 1 × 10^7^ CFU of TIGR4 or 2 × 10^6^ CFU of WCH43 in 50 µl PBS under deep isoflurane anaesthesia and culled after 24 or 48 h, respectively. Colonisation studies were performed using mice vaccinated intraperitoneally as outlined above and boosted intranasally under light isoflurane anaesthesia with 2 µg of the combination vaccines, 20 µl of diluted Prevnar-13 or PBS. Mice were infected intranasally with 1 × 10^7^ CFU of TIGR4 in 20 µl PBS under light isoflurane anaesthesia, culled 7 days post-infection, and degree of colonisation assessed by plating serial dilutions of nasal washes.^53^ Samples sizes were selected based on previous studies.^38,53^ No randomisation or blinding was performed. All animal procedures were approved by the local ethical review process and conducted in accordance with the relevant, UK Home Office approved, project license (PPL70/6510).

### Anti-capsule and anti-protein ELISAs

Anti-capsular responses were measured using Nunc Maxisorp plates were coated with 0.5 µg/well purified type 4 pneumococcal polysaccharide (Statens Serum Institut, Denmark) in PBS overnight at 4 °C, using the indicated dilutions of murine antiserum and a 1:20,000 dilution of horseradish peroxidase (HRP)-conjugated goat anti-mouse IgG (Abcam). Anti-capsule titres were determined by comparison with a standard curve generated using anti-Prevnar antiserum with an arbitrary titre of 1:10,000. Anti-protein responses were measured by sandwich ELISA using plates coated with 0.1 µg of monoclonal rabbit anti-His IgG (Abcam) and 1 µg/well of recombinant protein. For whole-cell ELISAs, TIGR4 and D39 cultures were grown to an OD_600_ of approximately 0.4–0.8, washed and resuspended to an OD_600_ of 0.4 in 10% glycerol, and plates incubated with 100 µl/well for 2 h at RT before fixation in 4% formaldehyde for 20 min. Plates were washed and incubated with a 1:1000 dilution of murine antiserum for 2 h at 28 °C using HRP-conjugated goat anti-mouse IgG for detection as outlined above.^53^

### Flow cytometry assays

Antibody deposition and neutrophil uptake assays were performed as previously described.^53^ Briefly, IgG binding was assessed using 2 × 10^6^ CFU bacteria in a final volume of 50 µl, detection with a 1:100 dilution of phycoerythrin (PE)-conjugated goat anti-mouse IgG (Thermo Fisher), and measurement of fluorescence intensity using a FACSVerse (BD Bioscience). TIGR4 deposition titres were determined by comparison with a standard curve, generated using anti-Prevnar antiserum with an arbitrary titre of 1:10,000. Neutrophils were purified from healthy human donors using the MACSxpress magnetic purification system according to the manufacturer’s instructions (Miltenyi Biotechnology), and 2 × 10^4^ neutrophils incubated for 30 min in a final volume of 50 µl per sample with 2 × 10^6^ FAM-SE labelled *S. pneumoniae* that had been pre-opsonised with diluted murine antiserum for 30 min at 37 °C. The reactions were fixed with 100 µl/well 4% paraformaldehyde and the fluorescence intensity of the neutrophil population was measured using the FACSVerse system. Experiments using human cells were approved by the joint University College London/University College Hospitals National Health Service Trust Human Research Ethics Committee, and informed consent was obtained from all participants.

### Fluorescence microscopy

Fluorescent microscopy was performed using formaldehyde fixed bacteria incubated with a 1:200 dilution of mouse antiserum and a 1:500 dilution of pneumococcal Omni serum (Statens Serum Institut) for 30 min at RT. PBS washed slides were incubated with a 1:10,000 dilution of Alexafluor488-conjugated anti-mouse IgG and Alexafluor568-conjugated anti-rabbit IgG for 15 min, incubated with a 1:10,000 dilution of 4',6-diamidino-2-phenylindole (DAPI) in PBS and mounted in DAKO mounting medium (Agilent). Fluorescent images were produced using an Olympus TIRF confocal microscope and Fluoview software (Olympus Lifesciences).

### Statistical analysis

Statistical analyses were performed using GraphPad Prism. ELISA data were analysed using one-way analysis of variance (ANOVA) with Bonferroni’s correction. Opsonisation, phagocytosis, and vaccine experiments were analysed using a Kruskal–Wallis with Dunn’s post-test. Flow cytometry data analysis was performed using FlowJo software.

## Electronic supplementary material

Supplementary Information

## Data Availability

All data generated or analysed during this study are included in this published article and its supplementary information files

## Electronic supplementary material

**Supplementary information** accompanies the paper on the *npj Vaccines* website (10.1038/s41541-018-0090-4).
