# Supplementary material for: A recombinant conjugated pneumococcal vaccine that protects against murine infections with a similar efficacy to Prevnar-13
Source: NPJ Vaccines. 2018 Oct 31;3:53. doi: 10.1038/s41541-018-0090-4 (PMC6208403; doi:10.1038/s41541-018-0090-4)
Supplement: Supplementary file 1 — Supplementary Information [file 41541_2018_90_MOESM1_ESM.pdf]

Supplementary Information

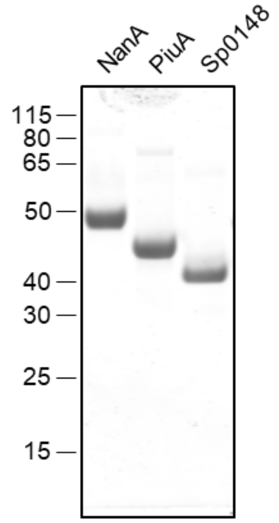

Figure S1: Visualisation of vaccine proteins to assess vaccine purity. Representative SDS-PAGE gel of purified recombinant vaccine proteins (2 µg/ well). Molecular mass markers are given in kilodaltons.

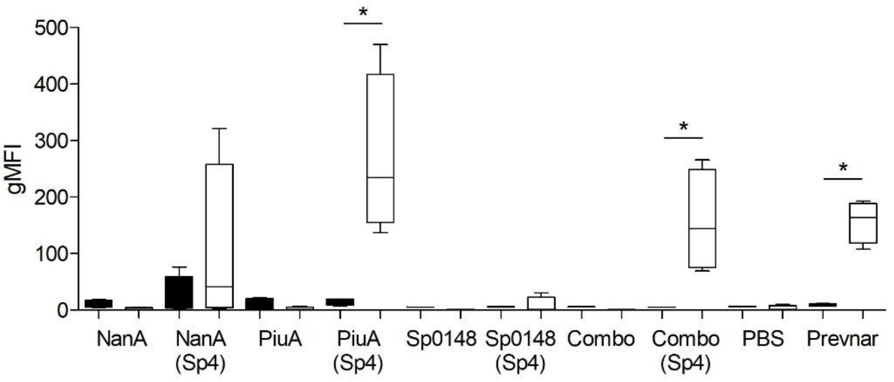

Figure S2: Antibody deposition on *Streptococcus mitis*. A) Antibody deposition measured using a flow cytometry assay on the *S. mitis* WT (black bars) and *S. mitis* expressing the type 4 pneumococcal capsule (White bars) in 10% murine antiserum (n=4). *p<0.05 two-tailed Students t-test (*S. mitis* WT vs *S. mitis* T4).

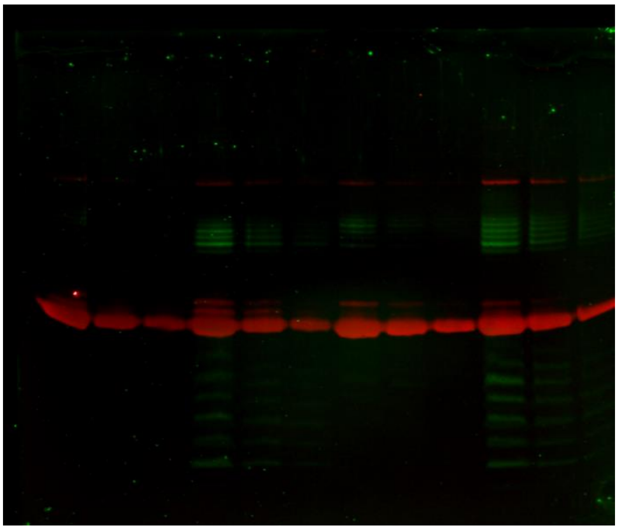

Figure S3: Full, uncropped blot from Figure 1A (Sp0148). No molecular weight marker was used.

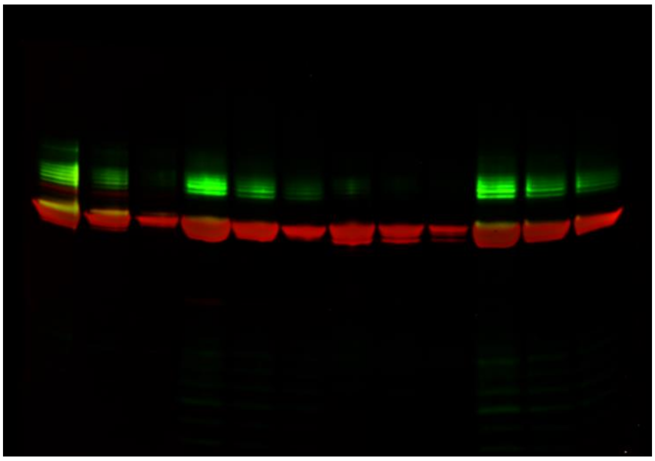

Figure S4: Full, uncropped blot from Figure 1B (NanA). No molecular weight marker was used.

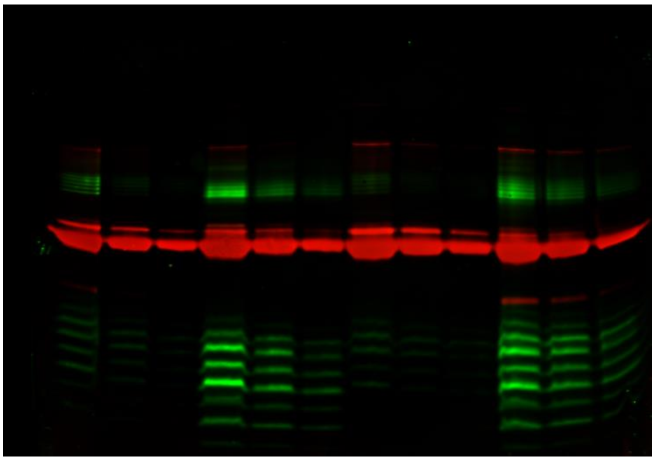

Figure S5: Full, uncropped blot from Figure 1C (PiuA). No molecular weight marker was used.

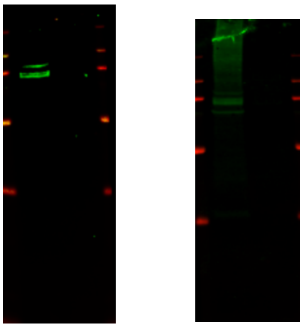

Figure S6: Full, uncropped blot from Figure 2F (NanA and NanA(Sp4)).

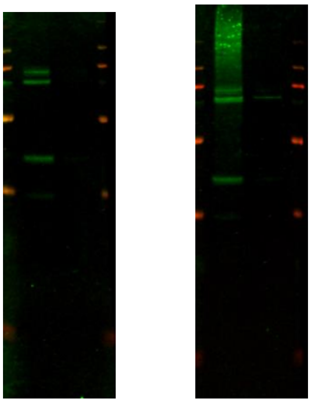

Figure S7: Full, uncropped blot from Figure 2F (PiuA and PiuA(Sp4)).

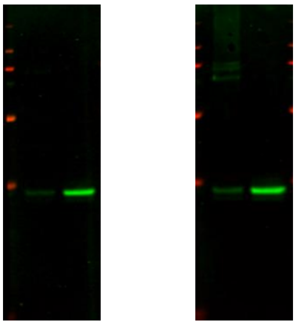

Figure S8: Full, uncropped blot from Figure 2F (Sp0148 and Sp0148(Sp4)).

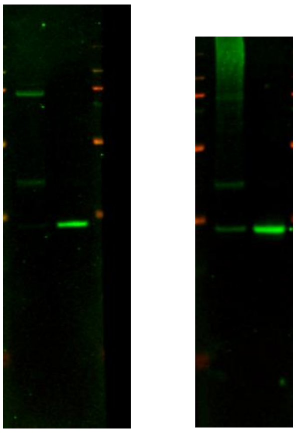

Figure S9: Full, uncropped blot from Figure 2F (Combo and Combo(Sp4)).

Supplementary methods

For recombinant protein production the N-terminal lectin-like domain of NanA was sub cloned from pEXT21 (nanA) using the primers listed in Table S3. Two fragments of the commercially synthesised NanA construct were amplified from pEXT21(nanA) using Q5 High-Fidelity 2X Master Mix (New England Biolabs) and the primer pairs pEXT-F/nanA-R and pEXT-R/nanA-F with the conditions: 98°C 10 s, 60°C 30 s, 72°C 30 s for 30 cycles. Fragments were PCR purified (QIAquick PCR Purification Kit, Qiagen) and digested with AvrII (New England Biolabs). Digested fragments were PCR purified and ligated using T3 DNA ligase (New England Biolabs). The religated reaction mixture was used as the template for NanA (N-terminus) amplification using the pEXT-F/pEXT-R primer pair as outlined above. The resulting insert was PCR purified and cloned into pEXT21 using the restriction enzymes EcoRI and XbaI. Plasmid pEXT21(NanA), containing the lectin-like domain of NanA and the genetic components required for PglB glycosylation and Ni-NTA purification, was transformed into DH5α cells and stored at -80°C in 20% glycerol.

To facilitate PglB glycosylation, synthetic carrier protein sequences were modified with the sequon DQNAT, flanked by two spacer glycine residues, at the N and C terminus of the mature protein. Periplasmic targeting was facilitated by exchanging native signal peptides for the DsbA leader sequence MKKIWLALAGLVLAFSASAAQ. C-terminal deca-His sequences were included to facilitate Ni-NTA purification. In *nanA*, internal EcoRI and XbaI sites were replaced with synonymous mutations. Commercial plasmid constructs were cloned into library efficient DH5α cells (Life Technologies) and stored at -80°C in 20% glycerol. Synthetic carrier protein sequences were sub cloned in the expression vector pEXT21 using the restriction enzymes EcoRI and XbaI (New England Biolabs).

Supplementary tables

Table S1: Strains used in this study

Species	Strain	Description
E. coli	W3110	K12 derivative
E. coli	W311B	W3110 derivative containing chromosomally inserted oligosaccharyltransferase PglB
E. coli	W3110 pB4-4	W3110 containing Spn. type 4 capsule operon
E. coli	W311B pB4-4	W311B containing Spn. type 4 capsule operon
E. coli	W3110 pEXT21(NanA)*#	recombinant NanA production
E. coli	W3110 pEXT21(PiuA)*	recombinant PiuA production
E. coli	W3110 pEXT21(Sp0148)*	recombinant Sp0148 production
E. coli	W311B pB4-4 pEXT21(NanA)	glycosylated NanA production
E. coli	W311B pB4-4 pEXT21(PiuA)	glycosylated PiuA production
E. coli	W311B pB4-4 pEXT21(Sp0148)	glycosylated Sp0148 production
E. coli	W311B pB4-4 pEXT20(GalE)	Enhanced Sp4 glycosylation
E. coli	W311B pB4-4 pEXT21(NanA) pEXT20 (galE)*	glycosylated NanA production
E. coli	W311B pB4-4 pEXT21(PiuA) pEXT20 (galE)*	glycosylated PiuA production
E. coli	W311B pB4-4 pEXT21(Sp0148) pEXT20 (galE)*	glycosylated Sp0148 production
S. pneumoniae	TIGR4	Serotype 4
S. pneumoniae	WCH43	Serotype 4 meningitis isolate [34]
S. pneumoniae	D39	Serotype 2
S. pneumoniae	EF3030	Serotype 19F
S. pneumoniae	6B	Serotype 6B
S. pneumoniae	23F	Serotype 23F
S. mitis	*S. mitis*	*S. mitis* expressing WT capsule
S. mitis	*S. mitis*(SpT4)	*S. mitis* expressing Spn. type 4 capsule[39]

*Used for vaccine production

#Recombinantly expresses an N-terminal fragment of NanA

Table S2: Synthetic carrier protein constructs. Ribosomal binding sites, start codons and stop codons are underlined. Glycosylation sequons are given in red. Synonymous mutations to remove internal restriction sites are highlighted in yellow.

NanA	GGATCCGAATTCCATTTTAAATAAGGAGGAATAACATAATGAAAAAGATTTGGCTGGCGCTGGCTGGTTTAGTTTTAGCGTTTAGCGCATCG<br>GCGGCGCAGGGAGGCGATCAGAACGCGACCGGTGGCGAGCAACCTCTGGCAAATGAAACTCAACTTTCGGGGGAGAGCTCAACCCTAACTGA<br>TACAGAAAAGAGCCAGCCTTCTTCAGAGACTGAACTTTCTGGCAATAAGCAAGAACAAGAAAGGAAAGATAAGCAAGAAGAAAAAATTCCAA<br>GAGATTACTATGCACGAGATTTGGAAAATGTCGAAACAGTGATAGAAAAAGAAGATGTTGAAACCAATGCTTCAAATGGTCAGAGAGTTGAT<br>TTATCAAGTGAACTAGATAAACTAAAGAAACTTGAAAACGCAACAGTTCACATGGAGTTTAAGCCAGATGCCAAGGCCCCAGCATTCTATAA<br>TCTCTTTTCTGTGTCAAGTGCTACTAAAAAAGATGAGTACTTCACTATGGCAGTTTACAATAATACTGCTACCCTAGAGGGGCGTGGTTCGG<br>ATGGGAAACAGTTTTACAATAATTACAACGATGCACCCTTAAAAGTTAAACCAGGTCAGTGGAACTCTGTGACTTTCACAGTTGAAAAACCG<br>ACAGCAGAACTACCTAAAGGCCGAGTGCGCCTCTACGTAAACGGGGTATTATCTCGAACAAGTCTGAGATCTGGCAATTTCATTAAAGATAT<br>GCCAGATGTAACGCATGTGCAAATCGGAGCAACCAAGCGTGCCAACAATACGGTTTGGGGGTCAAATCTACAGATTCGGAATCTCACTGTGT<br>ATAATCGTGCTTTAACACCAGAAGAGGTACAAAAACGTAGTCAACTTTTTAAACGCTCAGATTTAGAAAAAAAACTACCTGAAGGAGCGGCT<br>TTAACAGAGAAAACGGACATATTCGAAAGCGGGCGTAACGGTAAACCAAATAAAGATGGAATCAAGAGTTATCGTATTCCAGCACTTCTCAA<br>GACAGATAAAGGAACTTTGATCGCAGGTGCAGATGAACGCCGTCTCCATTCGAGTGACTGGGGTGATATCGGTATGGTCATCAGACGTAGTG<br>AAGATAATGGTAAAACTTGGGGTGACCGAGTAACCATTACCAACTTACGTGACAATCCAAAAGCTTCTGACCCATCGATCGGTTCACCAGTG<br>AATATCGATATGGTGTTGGTTCAAGATCCTGAAACCAAACGAATCTTTTCTATCTATGACATGTTCCCAGAAGGGAAGGGAATCTTTGGAAT<br>GTCTTCACAAAAAGAAGAAGCCTACAAAAAAATCGATGGAAAAACCTATCAAATCCTCTATCGTGAAGGAGAAAAGGGAGCTTATACCATTC<br>GAGAAAATGGTACTGTCTATACACCAGATGGTAAGGCGACAGACTATCGCGTTGTTGTAGATCCTGTTAAACCAGCCTATAGCGACAAGGGG<br>GATCTATACAAGGGTAACCAATTACTAGGCAATATCTACTTCACAACAAACAAAACTTCTCCATTTAGAATTGCCAAGGATAGCTATCTATG<br>GATGTCCTACAGTGATGACGACGGGAAGACATGGTCAGCGCCTCAAGATATTACTCCGATGGTCAAAGCCGATTGGATGAAATTCTTGGGTG<br>TAGGTCCTGGAACAGGAATTGTACTTCGGAATGGGCCTCACAAGGGACGGATTTTGATACCGGTTTATACGACTAATAATGTATCTCACTTA<br>AATGGCTCGCAATCTTCTCGTATCATCTATTCAGATGATCATGGAAAAACTTGGCATGCTGGAGAAGCGGTCAACGATAACCGTCAGGTAGA<br>CGGTCAAAAGATCCACTCTTCTACGATGAACAATAGACGTGCGCAAAATACAGAATCAACGGTGGTACAACTAAACAATGGAGATGTTAAAC<br>TCTTTATGCGTGGTTTGACTGGAGATCTTCAGGTTGCTACAAGTAAAGACGGAGGAGTGACTTGGGAGAAGGATATCAAACGTTATCCACAG<br>GTTAAAGATGTCTATGTTCAAATGTCTGCTATCCATACGATGCACGAAGGAAAAGAATACATCATCCTCAGTAATGCAGGTGGACCGAAACG<br>TGAAAATGGGATGGTCCACTTGGCACGTGTCGAAGAAAATGGTGAGTTGACTTGGCTCAAACACAATCCAATTCAAAAAGGAGAGTTTGCCT<br>ATAATTCGCTCCAAGAATTAGGAAATGGGGAGTATGGCATCTTGTATGAACATACTGAAAAAGGACAAAATGCCTATACCCTATCATTTAGA<br>AAATTTAATTGGGACTTTTTGAGCAAAGATCTGATTTCTCCTACCGAAGCGAAAGTGAAGCGAACTAGAGAGATGGGCAAAGGAGTTATTGG<br>CTTGGAGTTCGACTCAGAAGTATTGGTCAACAAGGCTCCAACCCTTCAATTGGCAAATGGTAAAACAGCACGCTTCATGACCCAGTATGATA<br>CAAAAACCCTCCTATTTACAGTGGATTCAGAGGATATGGGTCAAAAAGTTACAGGTTTGGCAGAAGGTGCAATTGAAAGTATGCATAATTTA<br>CCAGTCTCTGTGGCGGGCACTAAGCTTTCGAATGGAATGAACGGAAGTGAAGCTGCTGTTCATGAAGTGCCAGAATACACAGGCCCATTAGG<br>GACATCCGGCGAAGAGCCAGCTCCAACAGTCGAGAAGCCAGAATACACAGGCCCACTAGGGACATCCGGCGAAGAGCCAGCCCCGACAGTCG<br>AGAAGCCAGAATACACAGGCCCACTAGGGACAGCTGGTGAAGAAGCAGCTCCAACAGTCGAGAAGCCAGAATTTACAGGGGGAGTTAATGGT<br>ACAGAGCCAGCTGTTCATGAAATCGCAGAGTATAAGGGATCTGATTCGCTTGTAACTCTTACTACAAAAGAAGATTATACTTACAAAGCTCC<br>TCTTGCTCAGCAGGCACTTCCTGAAACAGGAAACAAGGAGAGTGACCTCCTAGCTTCACTAGGACTAACAGCTTTCTTCCTTGGTCTGTTTA<br>CGCTAGGGAAAAAGAGAGAACAAGGAGGCGATCAGAACGCGACCGGTGGCCATCACCATCATCACCATCATCACCATCATTAATTAAGTCTA<br>GAGGATCC
PiuA	GGATCCGAATTCCATTTTAAATAAGGAGGAATAACATAATGAAAAAGATTTGGCTGGCGCTGGCTGGTTTAGTTTTAGCGTTTAGCGCATCG<br>GCGGCGCAGGGAGGCGATCAGAACGCGACCGGTGGCGCCCTAGTGGCCAGCTTCTTGCTCCTACTTGGTGCATGTAGTACAAACTCAAGCAC<br>TAGTCAGACAGAGACCAGTAGCTCTGCTCCAACAGAGGTAACCATTAAAAGTTCACTGGACGAGGTCAAACTTTCCAAAGTTCCTGAAAAGA<br>TTGTGACCTTTGACCTCGGCGCTGCGGATACTATTCGCGCTTTAGGATTTGAAAAAAATATCGTCGGAATGCCTACAAAAACTGTTCCGACT<br>TATCTAAAAGACCTAGTGGGAACTGTCAAAAATGTTGGTTCTATGAAAGAACCTGATTTAGAAGCTATCGCCGCCCTTGAGCCTGATTTGAT<br>TATCGCTTCGCCACGTACACAAAAATTCGTAGACAAATTCAAAGAAATCGCCCCAACCGTTCTCTTCCAAGCAAGCAAGGACGACTACTGGA<br>CTTCTACCAAGGCTAATATCGAATCCTTAGCAAGTGCCTTCGGCGAAACTGGTACACAGAAAGCCAAGGAAGAATTGACCAAGCTAGACAAG<br>AGCATCCAAGAAGTCGCTACTAAAAATGAAAGCTCTGACAAAAAAGCCCTTGCGATCCTCCTTAATGAAGGAAAAATGGCAGCCTTTGGTGC<br>CAAATCTCGTTTCTCTTTCTTGTACCAAACCTTGAAATTCAAACCAACTGATACAAAATTTGAAGACTCACGCCACGGACAAGAAGTCAGCT<br>TTGAAAGTGTCAAAGAAATCAACCCTGACATCCTCTTTGTCATCAACCGTACCCTTGCCATCGGTGGGGACAACTCTAGCAACGACGGTGTC<br>CTAGAAAATGCCCTTATCGCTGAAACACCTGCTGCTAAAAATGGTAAGATTATCCAACTAACACCAGACCTCTGGTATCTAAGCGGAGGCGG<br>ACTTGAATCAACAAAACTCATGATTGAAGACATACAAAAAGCTTTGAAAGGAGGCGATCAGAACGCGACCGGTGGCCATCACCATCATCACC<br>ATCATCACCATCATTAATTAAGTCTAGAGGATCC
Sp0148	GGATCCGAATTCCATTTTAAATAAGGAGGAATAACATAATGAAAAAGATTTGGCTGGCGCTGGCTGGTTTAGTTTTAGCGTTTAGCGCATCG<br>GCGGCGCAGGGAGGCGATCAGAACGCGACCGGTGGCGCAGCCCTTGCTCTTGTTGCTGCAGGTGTGCTTGCGGCTTGCTCAGGGGGTGCTAA<br>GAAAGAAGGAGAAGCAGCTAGCAAGAAAGAAATCATCGTTGCAACCAATGGATCACCAAAGCCATTTATCTATGAAGAAAATGGCGAATTGA<br>CTGGTTACGAGATTGAAGTCGTTCGCGCTATCTTTAAAGATTCTGACAAATATGATGTCAAGTTTGAAAAGACAGAATGGTCAGGTGTCTTT<br>GCTGGTCTTGACGCTGATCGTTACAATATGGCTGTCAACAATCTTAGCTACACTAAAGAACGTGCGGAGAAATACCTCTATGCCGCACCAAT<br>TGCCCAAAATCCTAATGTCCTTGTCGTGAAGAAAGATGACTCTAGTATCAAGTCTCTCGATGATATCGGTGGAAAATCGACGGAAGTCGTTC<br>AAGCCACTACATCAGCTAAGCAGTTAGAAGCATACAATGCTGAACACACGGACAACCCAACTATCCTTAACTATACTAAGGCAGACTTGCAA<br>CAAATCATGGTACGTTTGAGCGATGGACAATTTGACTATAAGATTTTTGATAAAATCGGTGTTGAAACAGTGATCAAGAACCAAGGTTTGGA<br>CAACTTGAAAGTTATCGAACTTCCAAGCGACCAACAACCGTACGTTTACCCACTTCTTGCTCAGGGTCAAGATGAGTTGAAATCGTTTGTAG<br>ACAAACGCATCAAAGAACTTTATAAAGATGGAACTCTTGAAAAATTGTCTAAACAATTCTTCGGAGACACTTATCTACCGGCAGAAGCTGAT<br>ATTAAAGGAGGCGATCAGAACGCGACCGGTGGCCATCACCATCATCACCATCATCACCATCATTAATTAAGTCTAGAGGATCC

Table S3 primers used in this study. AvrII restriction sites are underlined.

NanA-F		TTTCCTAGGACGCTAGGGAAAAAGAGAGAACAAGGAG
NanA-R		TTTCCTAGGCTCTGTTAAAGCCGCTCCTTCAGGTAG
pEXT-R		CGACGAATTTCTTCTCTCAT
pEXT-F		AGCGGATAACAATTTCACAC
